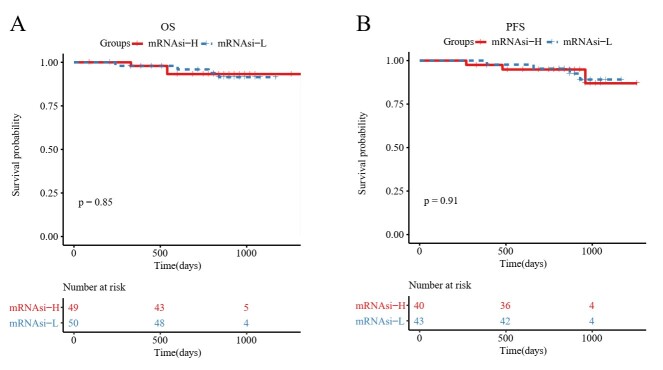# Identification of key genes regulating colorectal cancer stem cell characteristics by bioinformatics analysis

**DOI:** 10.1097/MD.0000000000040910

**Published:** 2025-05-30

**Authors:** Jing Lu, Haotian Zhang, Xiaoyu Gu, Yonghui Liu, Chengwen Zhao, Xudong Wang

**Affiliations:** a Medical School of Nantong University, Nantong, China; b Department of Laboratory Medicine, Affiliated Hospital of Nantong University, Nantong, China; c Department of Laboratory Medicine, Dongtai People’s Hospital, Yancheng, China; d Center of Clinical Laboratory Medicine, Zhongda Hospital, Southeast University, Nanjing, China; e Center of Clinical Laboratory Medicine, XuZhou Central Hospital, Xuzhou, China.

**Keywords:** cancer stem cells, colorectal cancer, differentially expressed genes, mRNA expression-based stemness index, The Cancer Genome Atlas, therapeutic targets

## Abstract

Cancer stem cells (CSCs), distinguished by their abilities to differentiate and self-renew, play a pivotal role in the progression of colorectal cancer (CRC). However, the mechanisms that sustain CSCs in CRC remain unclear. This study aimed to identify and characterize gene expressions associated with CRC stemness. We applied a 1-class logistic regression machine learning model to calculate the mRNA expression-based stemness index (mRNAsi) for CRC samples from The Cancer Genome Atlas and cBioPortal databases, adjusting the mRNAsi by tumor purity. Clinical features of CRC were considered in assessing both mRNAsi and adjusted mRNAsi levels. Using DESeq2, we screened differentially expressed genes between high and low mRNAsi groups. Enrichment analysis provided functional annotation for these differentially expressed genes. Key genes linked to mRNAsi were identified using the Kaplan–Meier plotter and Cytoscape software, followed by an evaluation of their prognostic significance. Potential small-molecule compounds targeting the CRC stemness signature were explored via L1000FWD, DGIdb, and CMap databases. CRC samples with higher mRNAsi or adjusted mRNAsi values showed improved disease-free survival (DSS) and progression-free survival (PFS). Strong correlation between clinical characteristics of CSCs and mRNAsi was observed; CMS4 subtype CRC patients had lower mRNAsi with worse DSS and PFS. Ten key genes associated with mRNAsi were identified: collagen type I alpha 1, fibrillin 1, matrix metalloproteinase 9, SPP1, BGN, COL5A1, FN1, elastin, matrix metalloproteinase 2, collagen type I alpha 2. Lower expression of these genes correlated with better PFS and DSS. High correlation among these genes was confirmed in the protein–protein interaction network. This study identifies potential small-molecule drugs targeting stemness in CRC and highlights the prognostic value of the 10 key genes, offering insights into therapeutic targets for CRC treatment.

## 1. Introduction

Colorectal cancer (CRC) ranks as the second leading cause of cancer-related mortality worldwide and is the third most common cancer, highlighting its major impact on global health and the need for enhanced understanding of its underlying mechanisms.^[1]^ The introduction of gene expression profiling has been instrumental in CRC research, leading to the definition of biologically distinct consensus molecular subgroups (CMS) and establishing a widely accepted classification of CRC into 4 molecular subgroups.^[2,3]^ Each subgroup exhibits unique biological characteristics and clinical outcomes, making them essential for understanding disease heterogeneity and guiding tailored therapeutic approaches. CMS1 features high immune infiltration and microsatellite instability (MSI), indicating a unique response to immunotherapy.^[4]^ CMS2 is marked by strong activation of Wingless/Integrated and myelocytomatosis oncogen signaling pathways, important for cell growth. CMS3 is linked to epithelial and metabolic issues, suggesting a specific metabolic profile and potential targets for therapy. CMS4 includes mesenchymal-like cancers, characterized by transforming growth factor-β pathway activation, stromal invasion, and angiogenesis, leading to a more aggressive tumor type. Despite the significant improvements in patient survival through the use of nonspecific multimodal therapies, such as radiotherapy, chemotherapy, and surgery, the 5-year relative survival rate for CRC patients remains unacceptably low. This underscores the urgent need for a more systematic exploration of the molecular mechanisms driving CRC progression and colorectal carcinogenesis, as understanding these processes may reveal critical CRC-related genes and signaling pathways, facilitating the discovery of more effective therapeutic strategies.

With advancements in single-cell RNA sequencing technology, the complexity of tumor heterogeneity has become more comprehensively understood. These advancements have revealed that tumors are not merely composed of cancer cells but represent a diverse and integrated ecosystem, including various supporting cells such as tumor-associated fibroblasts, cancer stem cells (CSCs), differentiated tumor cells, infiltrating immune cells, and endothelial cells.^[5]^ Within this ecosystem, CSCs represent a unique subpopulation of tumor cells with stem cell (SC)-like properties, allowing them to self-renew, differentiate, and generate a phenotypically diverse tumor cell population.^[6]^ Although CSCs make up only a small, tumorigenic subset, they are increasingly regarded as primary drivers of critical aspects of tumor behavior, including initiation, metastasis, recurrence, and resistance to conventional therapies.^[7]^ CSCs demonstrate a high degree of cellular flexibility, resulting in distinct roles, cellular phenotypes, and metabolic signatures within the tumor microenvironment.^[8]^ Emerging evidence supports the notion that human cancer may be an SC-driven disease, with CSCs closely linked to colorectal carcinogenesis.^[9]^ Lineage-tracing experiments have confirmed the presence of CSCs in CRC,^[10]^ establishing both a functional model for CSCs in CRC and a robust surface marker profile for their identification and isolation.^[11]^ Moreover, targeted strategies against CSCs have shown promising effectiveness in treating CRC, suggesting a potential therapeutic avenue for overcoming traditional treatment limitations.^[12]^ However, a comprehensive understanding of CRC stemness remains limited, especially in terms of the specific role of CSCs in cancer pathophysiology and the identification of key genes or pathways that drive CSCs toward a malignant state.

In recent years, machine learning, a branch of artificial intelligence and deep learning, has become widely used in the field of medical and biological data mining, enabling new approaches to data interpretation and discovery. The 1-class logistic regression (OCLR) algorithm, introduced in 2016, is a specific machine learning technique designed to classify molecular cell types by deconvoluting RNA sequencing data.^[13]^ In a seminal study, Malta et al^[5]^ leveraged the innovative OCLR method to extract transcriptomic and epigenetic data from pluripotent SCs and their differentiated progeny. Their approach facilitated the development of 2 stemness indices: DNA methylation-based stemness index and mRNA expression-based stemness index (mRNAsi), enabling a multi-platform analysis for identifying SC features and quantifying stemness.^[5]^ The introduction of these indices has offered a valuable tool for SC research, allowing researchers to quantitatively assess stemness and providing insights into the molecular characteristics that define stem-like properties in cancer cells.

In this study, our objective was to identify key genes and pathways associated with CRC stemness by leveraging CRC samples from The Cancer Genome Atlas (TCGA) (n = 521) and cBioPortal (n = 110) datasets. To achieve this, we constructed a stemness index model using the OCLR algorithm to quantify COAD stemness,^[14]^ resulting in an independent stemness index termed mRNAsi. However, the underlying mechanisms and clinical significance of mRNAsi in CRC remain largely unclear. We then conducted a comprehensive analysis to assess the associations between the stemness index and various clinical outcomes, including survival curves, as well as other clinical and molecular features. Our findings revealed significant correlations, suggesting that the stemness index could serve as a potential marker for disease prognosis and treatment responsiveness. Additionally, we identified a list of stemness-related genes that may play essential roles in CRC biology, thereby underscoring their importance as potential therapeutic targets. Lastly, by utilizing multiple databases, including L1000FWD, DGIdb, and CMap,^[15–17]^ we identified specific compounds that target CRC stemness signatures. These compounds offer promising potential avenues for the development of novel CRC treatments, aimed at improving patient outcomes by targeting the underlying mechanisms of CRC stemness. This study, therefore, contributes to a deeper understanding of CRC stemness, paving the way for new therapeutic strategies focused on targeting CSCs and improving CRC patient survival.

## 2. Materials and methods

### 2.1. Data collection and processing

RNA sequencing (RNA-Seq) expression data for 521 COAD samples, along with corresponding clinical information, was downloaded from TCGA database in September 2019 (https://portal.gdc.cancer.gov/). Additionally, RNA-Seq data and clinical information for 110 COAD samples were obtained from the Colon Cancer dataset (CPTAC-2 Prospective, Cell 2019) in the cBioPortal database in November 2019 (http://www.cbioportal.org), hereafter referred to as CPTAC-2.^[18]^ Gene expression array data for pluripotent SC samples was also downloaded from the Progenitor Cell Biology Consortium dataset using the R package “synapser” (v0.6.61) in November 2019.^[19,20]^ For the TCGA-COAD dataset, samples with gene expression data were retained, and Ensembl IDs were annotated as Gene Symbols, preserving only protein-coding genes. Additionally, gene expression profiles for samples from primary solid tumor and solid tissue normal were included. For the CPTAC-2 dataset, samples with gene expression data were retained, and survival times were standardized in days. A summary of the statistical details is provided in Table 1.

**Table 1:** Clinical features of CRC patients in TCGA and CPTAC-2 datasets.

Clinical features	TCGA-COAD	CPTAC-2
Type		
Normal	41	0
Tumor	454	106

OS/DSS	DSS	OS
Alive	352	91
Dead	59	8

OS/DSS time (mean)	DSS	OS
Alive	962.3438	864.3956
Dead	569.661	648.75
PFS		
Alive	310	76
Dead	114	7
PFS time (mean)		
Alive	925.7065	892.5
Dead	524.4123	651.4286
T stage		
T1	11	
T2	77	16
T3	309	76
T4	56	14
TX	1	
N stage		
N0	267	56
N1	105	34
N2	0	16
NX	82	
M stage		
M0	333	
M1	64	
MX	57	
Stage		
I	75	12
II	176	42
III	128	45
IV	64	7
X	11	
Lymphatic invasion		
Yes	99	
No	82	
Unknown	273	
Vascular invasion		
Yes	44	
No	122	
Unknown	288	
Methylation subtype		
CIMP.H	33	
CIMP.L	38	
Cluster3	47	
Cluster4	46	
Unknown	290	
MSI status		
MSI-H	37	24
MSI-L	40	0
MSS	121	81
Unknown	256	1
CMS		
CMS1	82	17
CMS2	133	33
CMS3	68	18
CMS4	131	33
Unknown	40	5
Phenotype		
EMT		25
Epithelial		60
Hypermutated		21

CMS = consensus molecular subgroups, CRC = colorectal cancer, DSS = disease-free survival, EMT = epithelial-mesenchymal transition, MSI = microsatellite instability, OS = overall survival, PFS = progression-free survival, TCGA = The Cancer Genome Atlas.

### 2.2. Calculation of gene expression-based stemness indices for CRC

Embryonic stem cells and induced pluripotent stem cell samples from the progenitor cell biology consortium dataset were retained as SC samples. Ensembl IDs were converted to Gene Symbols, and only protein-coding genes were preserved. This resulted in 78 SC samples, each with a gene expression profile consisting of 12,998 genes. The data were mean-centered, and the R package “gelnet” was used to calculate the weight vector for each gene. To determine stemness indices, we computed Spearman correlations between the gene expression profiles and the weight vector for TCGA-COAD samples. A linear transformation was applied to map these indices to a [0, 1] range by subtracting the minimum Spearman correlation and dividing by the maximum. This method can also be applied to CPTAC-2 samples. The mRNAsi values were generated using log2-transformed normalized gene expression data; the full procedure for calculating mRNAsi is available at https://bioinformaticsfmrp.github.io/PanCanStem_Web/.

### 2.3. Clinical characteristic correlation analysis

Samples were divided into 2 groups based on the median mRNAsi value: mRNAsi-L (values below the median) and mRNAsi-H (values equal to or above the median). Using RNA-Seq data from the TCGA-COAD and CPTAC-2 datasets, we analyzed mRNAsi values across different CMS molecular subtypes with the R package “CMScaller” (v0.99.1), a tool designed for CMS classification without reliance on human tumor stroma.^[21]^ Additionally, mRNAsi values were examined across various clinical characteristics, including tumor stages, tumor node metastasis (TNM) subsets, MSI status, methylation subtypes, and phenotypes, using the R package “TCGAbiolinks” (v2.14.0).^[22]^ We further assessed correlations between mRNAsi and progression-free survival (PFS) and disease-free survival (DSS) through Kaplan–Meier survival plots, using the R survival package. Criteria for excluding samples from survival analysis included: samples lacking survival time or survival status, and samples with survival time <30 days. A *P* value of <.05 was considered statistically significant.

### 2.4. Identification of differentially expressed genes

Differentially expressed genes (DEGs) between mRNAsi-H and mRNAsi-L samples in the TCGA-COAD dataset were identified using the R package “DESeq2” (v1.24.0). DEGs from the CPTAC-2 dataset were identified using the “limma” package in R.^[23]^ Selection criteria for DEGs were set as follows: false discovery rate <0.05 and |log2 fold change| >1. Volcano plots of the DEGs were generated using the “ggplot2” and “ggrepel” packages.

### 2.5. GO and Kyoto Encyclopedia of Genes and Genomes pathway enrichment analyses of DEGs

Gene ontology (GO) function enrichment analysis was conducted using the “clusterProfiler” package (v3.14.0), an ontology-based R package used to categorize biological themes and provide functional interpretations for gene clusters based on TCGA-COAD and CPTAC-2 datasets. Additionally, the Kyoto Encyclopedia of Genes and Genomes (KEGG) pathway enrichment was performed using the same package.^[24]^ GO terms and KEGG pathways with a false discovery rate <0.05 were considered significantly enriched.

### 2.6. Protein–protein interaction network construction and hub gene selection

The Search Tool for the Retrieval of Interacting Genes database (http://string-db.org) was used to construct the protein–protein interaction (PPI) network for DEGs, with a combined score cutoff >0.4.^[25]^ The PPI network was visualized using Cytoscape (version 3.7.2) to explore interaction networks, and the top hub genes were identified with the CytoHubba plugin. The top 10 ranked genes were selected as hub genes.

### 2.7. Prognosis analysis of key genes

Pearson correlation analysis was conducted to assess mutual correlations between hub genes and stemness indices. The associations between mRNAsi values and gene expression levels were evaluated using the Pearson Correlation test. Additionally, we analyzed the survival significance of key genes using the online database (http://www.kmplot.com/).

### 2.8. Identification of potential compounds targeting the CRC stemness signature

The L1000 Fireworks Display (L1000FWD; http://amp.pharm.mssm.edu/L1000FWD) is a high-throughput gene expression profiling platform that provides gene expression signatures from drug-induced cancer cell lines, covering over 16,000 drugs. The Drug-Gene Interaction Database (DGIdb; http://www.dgidb.org/search_interactions) was used to integrate gene–drug interactions and identify potential drug-gene susceptibilities from various data sources.

Additionally, we utilized connectivity Map (CMap; www.broad.mit.edu/cmap), a database containing more than 1309 compounds and 7000 gene expression profiles from cancer cell lines treated with small-molecule drugs. By querying an interested gene expression profile against CMap data, a list of compounds can be scored using a pattern-matching algorithm. A positive connectivity score suggests the drug may promote cancer, while a negative score indicates the drug may counteract cancer progression. Compounds with a negative connectivity score were considered potential therapeutic agents.

### 2.9. Statistical analysis

All data processing and statistical analyses were conducted using R 3.6.1 (https://cran.r-project.org). Survival differences between the 2 groups were evaluated using a 2-sided log-rank test. The Wilcoxon test and Kruskal–Wallis test functions in R were used to assess the correlation between mRNAsi values and clinical characteristics. A *P* value <.05 was considered statistically significant.

## 3. Results

### 3.1. Prognostic value of mRNAsi in CRC

**Figure 1.**
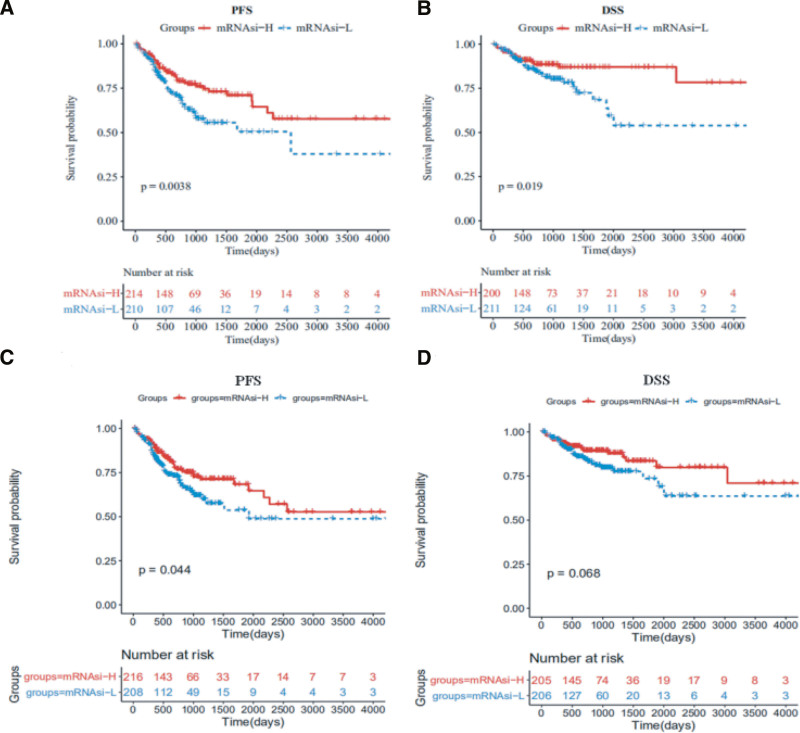
The correlation between mRNAsi and PFS and DSS in CRC patients for TCGA database. (A, B) Kaplan–Meier curves show that the high mRNAsi group had better PFS and DSS than the low mRNAsi group. (C, D) Kaplan–Meier curves show that the higher corrected mRNAsi group had better PFS and DSS than that of the lower corrected mRNAsi group. CRC = colorectal cancer, DSS = disease-free survival, mRNAsi = mRNA expression-based stemness index, PFS = progression-free survival, TCGA = The Cancer Genome Atlas.

To calculate the mRNAsi, we established a predictive model using the OCLR algorithm to train a stemness signature on sample data. CRC samples were then divided into mRNAsi-L and mRNAsi-H groups, and Kaplan–Meier curves were constructed to evaluate the association between mRNAsi levels and survival probabilities. For samples from the TCGA database, those with higher mRNAsi values exhibited significantly better PFS and DSS compared to those with lower mRNAsi values (Fig. 1A and B). However, in the CPTAC-2 database, differences in overall survival and PFS were less pronounced, likely due to a smaller sample size and a shorter observation period (Figure S1, Supplemental Digital Content, http://links.lww.com/MD/O184). The mRNAsi index, derived from molecular profiles of normal cells with varying degrees of stemness, can be influenced by the heterogeneity of tumor tissue, which consists of various cell types. This prompted us to account for tumor purity by calculating a corrected mRNAsi (mRNAsi/tumor purity). With this adjustment, we observed similar results: CRC samples with higher corrected mRNAsi values had improved PFS and DSS (Fig. 1C and D). Although these findings differ from the general understanding of CSC characteristics, they align with the work of Malta et al^[5]^ regarding CRC, suggesting that the mRNAsi index calculated via the OCLR method is closely related to cancer type.

### 3.2. mRNAsi and corrected mRNAsi according to clinical characteristics of CRC

**Figure 2.**
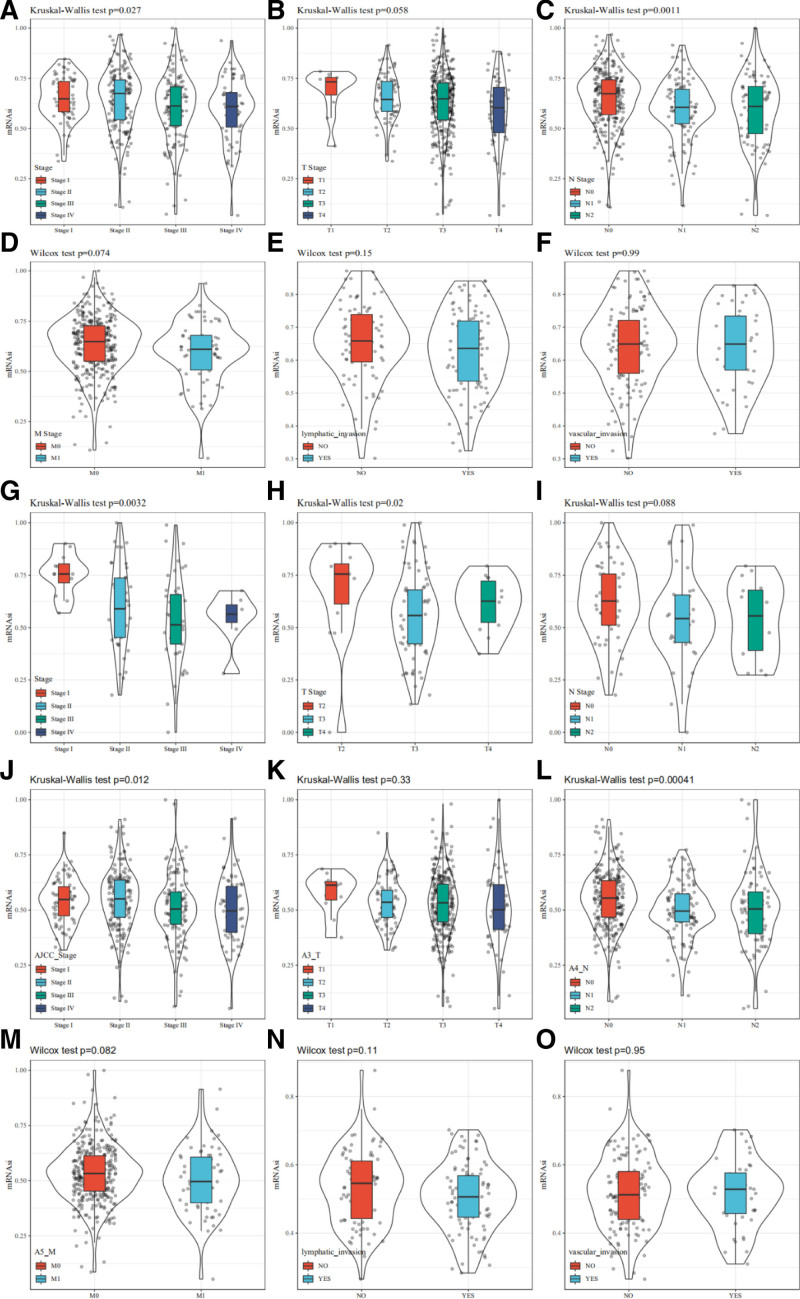
Comparison between mRNAsi values and clinical characteristics in CRC for TCGA database, including clinical stage (A), TNM stage (B–D), lymphatic invasion (E), and vascular invasion (F). Comparison between mRNAsi values and clinical characteristics in CRC for CPTAC-2 database, including (G) clinical stage, (H) T stage, and (I) N stage. Comparison between corrected mRNAsi values and clinical characteristics in CRC for TCGA database, including (J) clinical stage, (K–M) TNM stage, (N) lymphatic invasion, and (O) vascular invasion. CRC = colorectal cancer, mRNAsi = mRNA expression-based stemness index, TCGA = The Cancer Genome Atlas.

Using the TCGA database, we investigated whether mRNAsi values varied across different clinical characteristics of CRC, including clinical stage, TNM stage, lymphatic invasion, and vascular invasion. For clinical stage, we observed a significant variation in mRNAsi values across pathologic stages, with a trend of gradual decrease (*P* = .027, Fig. 2A). Regarding TNM staging, mRNAsi values differed significantly across Node stages (*P* = .0011, Fig. 2C). Although mRNAsi values showed a decreasing trend across tumor stages, metastasis stages, and lymphatic invasion subsets, these differences were not statistically significant (*P* > .05, Fig. 2B, D, and E). Additionally, while mRNAsi was higher in samples with vascular invasion, this increase was not statistically significant (*P* > .05, Fig. 2F). Similarly, in the CPTAC-2 database, distinct pathologic stages showed significant differences in mRNAsi values, with a decline as stages advanced (*P* = .032, Fig. 2G). For tumor staging, mRNAsi values for T3 and T4 stages were significantly lower than those for T2 (*P* = .02, Fig. 2H), although node stages did not show significant differences in mRNAsi (*P* > .05, Fig. 2I). Given the potential influence of tumor purity, we reevaluated these characteristics using the corrected mRNAsi, yielding similar results (Fig. 2J–O). These findings indicate that both mRNAsi and corrected mRNAsi are closely associated with the clinical characteristics of CSCs in CRC.

### 3.3. Correlations between stemness indices and molecular subtypes

**Figure 3.**
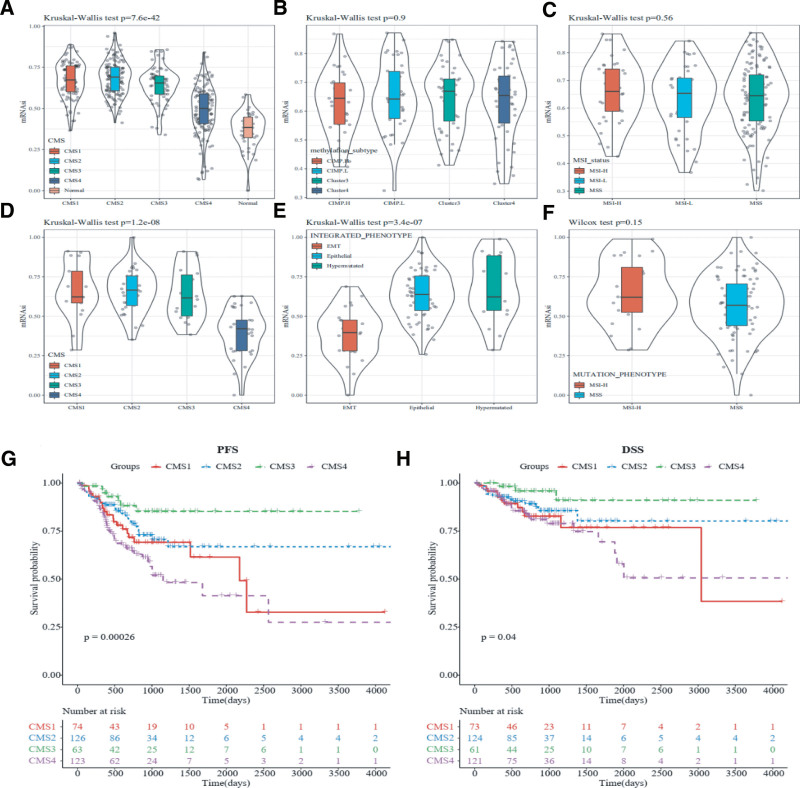
mRNAsi stratified by different molecular subtypes in TCGA and CPTAC-2 datasets, including (A, D) CMS subtypes, (B, E) methylation subtypes, (C, F) MSI status. K–M curves showing correlations between molecular subtypes and PFS and DSS in CRC patients. (G) PFS between 4 CMS subtypes in CRC patients. (H) DSS between 4 CMS subtypes in CRC patients. CMS = consensus molecular subgroups, CRC = colorectal cancer, DSS = disease-free survival, K–M = Kaplan–Meier, MSI = microsatellite instability, PFS = progression-free survival, TCGA = The Cancer Genome Atlas.

We compared mRNAsi values across CMS molecular subtypes, MSI status, methylation subtypes, and phenotypes. For the TCGA database, mRNAsi values were significantly higher in the 4 distinct CMS molecular subtypes than in normal samples (*P* < 2.2e-16, Fig. 3A). However, mRNAsi values did not differ significantly across methylation subtypes and MSI statuses (*P* > .05, Fig. 3B and C). In the CPTAC-2 database, significant differences in mRNAsi values were observed among the CMS molecular subtypes (*P* = 1.2e-08, Fig. 3D) as well as among phenotypes (epithelial-mesenchymal transition [EMT], epithelial, and hypermutated subtypes) (*P* = 3.4e-07, Fig. 3E). Additionally, we analyzed mRNAsi values between the MSI-H and microsatellite stability groups, as MSI-L samples were not available, and found no significant difference between the 2 groups (*P* > .05, Fig. 3F). Given that patients with the CMS4 subtype showed significantly lower mRNAsi values compared to the other CMS subtypes in both the TCGA-COAD and CPTAC-2 databases, we conducted Kaplan–Meier survival analyses. The results indicated that patients with the CMS4 subtype had significantly worse PFS and DSS than those with other subtypes (Fig. 3G and H).

### 3.4. Identification, gene function annotation, and pathway analysis of DEGs

**Figure 4.**
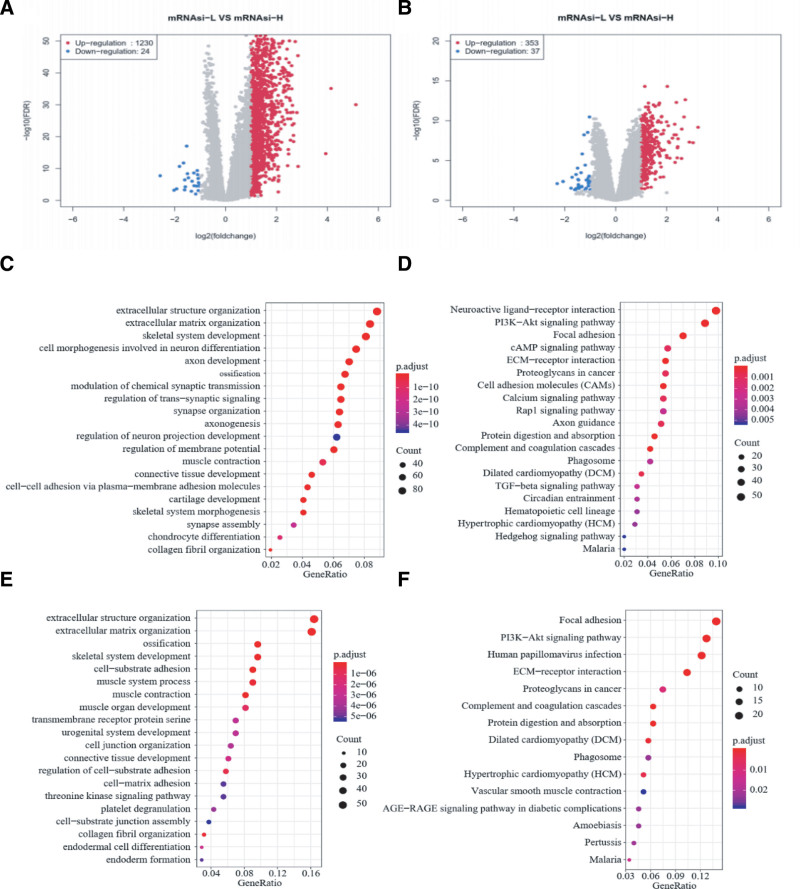
Volcano plot of the DEGs between the mRNAsi-H and mRNAsi-L groups. (A) Volcano plot showing differentially expressed genes in TCGA database. (B) Volcano plot showing differentially expressed genes in CPTAC-2 database. The red point represents the up-regulated genes; the blue point represents the down-regulated genes GO and KEGG enrichment analyses of DEGs. (C) GO enrichment analysis of DEGs in TCGA-COAD dataset. (D) KEGG pathway enrichment analysis of DEGs in TCGA-COAD dataset. (E) GO enrichment analysis of DEGs in CPTAC-2 dataset. (F) KEGG pathway enrichment analysis of DEGs in CPTAC-2 dataset. The count of DEGs enriched under each term is indicated by the size of dots. The color of the dot represents *P* value. DEG = differentially expressed gene, GO = gene ontology, KEGG = Kyoto Encyclopedia of Genes and Genomes, mRNAsi = mRNA expression-based stemness index, TCGA = The Cancer Genome Atlas.

To investigate the functional genes associated with mRNAsi, we analyzed the TCGA database using R software. A total of 1254 genes were identified, with 1230 up-regulated and 24 down-regulated in the mRNAsi-L group compared to the mRNAsi-H group (Fig. 4A). Similarly, in the CPTAC-2 dataset, 390 genes were identified, including 353 up-regulated and 37 down-regulated in the mRNAsi-L group (Fig. 4B). This indicates a pronounced upregulation of genes in the mRNAsi-L group. Volcano plots illustrate that the majority of DEGs were up-regulated, with 221 up-regulated genes overlapping between the 2 datasets. Notably, there was no overlap among down-regulated genes across the datasets. To further understand the biological characteristics of these DEGs, we performed GO and KEGG pathway enrichment analyses using the “clusterProfiler” R package. GO analysis encompassed 3 categories: molecular function, cellular component, and biological process. For the TCGA and CPTAC-2 datasets, DEGs were enriched in 1077 and 498 GO terms, respectively. As shown in Figure 4C–F, the DEGs were primarily associated with extracellular structure organization, extracellular matrix (ECM) organization, and ossification. In the KEGG pathway enrichment analysis, DEGs were mainly associated with focal adhesion, PI3K-Akt signaling pathway, and ECM-receptor interaction, suggesting these pathways play a significant role in mRNAsi-related processes in CRC.

### 3.5. Construction of PPI network for hub genes in CRC

**Figure 5.**
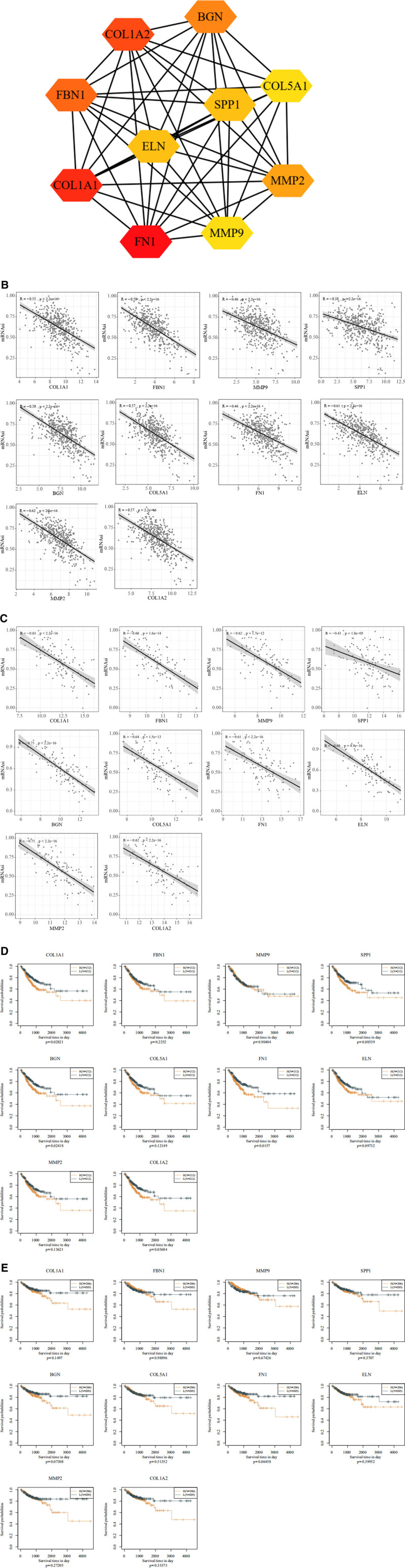
Construction of PPI network and correlations between expression levels of 10 hub genes and mRNAsi values in TCGA and CPTAC-2 datasets. (A) The number of the solid line indicates the strength of the relationship. (B) The expression levels of 10 hub genes were negatively related to mRNAsi values in TCGA database. (C) The expression levels of 10 hub genes were negatively related to mRNAsi values in CPTAC-2 database. (D) PFS of low and high expression of key genes in Kaplan–Meier plotter database. (E) DSS of low and high expression of key genes in Kaplan–Meier plotter database. DSS = disease-free survival, mRNAsi = mRNA expression-based stemness index, PFS = progression-free survival, PPI = protein–protein interaction, TCGA = The Cancer Genome Atlas.

Using the Cytohubba plugin in Cytoscape, we identified the top 10 hub genes for further analysis: collagen type I alpha 1 (COL1A1), fibrillin 1, matrix metalloproteinase 9, secreted phosphoprotein 1, biglycan‌‌ (BGN), collagen, type V, alpha 1, fibronectin 1 (FN1), elastin, matrix metalloproteinase 2, and collagen type I alpha 2 (COL1A2). To examine the interactions among these genes at the protein level, we constructed a PPI network via the Search Tool for the Retrieval of Interacting Genes database, which revealed strong associations between the hub genes (Fig. 5A). Pearson correlation analysis and Kaplan–Meier survival analysis were conducted to evaluate the prognostic potential of these hub genes. As shown in Figure 5B and C, the expression levels of the 10 hub genes were significantly negatively correlated with mRNAsi in both the TCGA-COAD and CPTAC-2 datasets. Additionally, survival analysis indicated that CRC patients with low expression levels of these genes had better PFS and DSS. In particular, the expression of COL1A1, COL1A2, BGN, and FN1 had a significant impact on the PFS of CRC patients (Fig. 5D and E). These findings suggest that our study’s approach is effective for identifying key genes associated with CRC stemness indices.

### 3.6. Identification of potential small-molecule drugs

**Figure 6.**
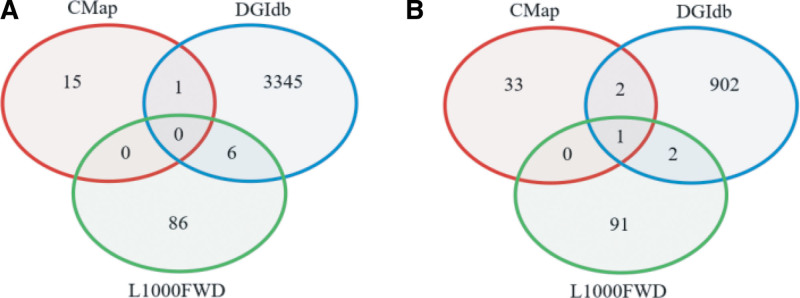
Compounds overlapped in the 3 databases for TCGA and CPTAC-2 datasets. (A) Compounds overlapped in the 3 databases for TCGA database. (B) Compounds overlapped in the 3 databases for CPTAC-2 database. TCGA = The Cancer Genome Atlas.

For the TCGA dataset, we uploaded the 1254 DEGs, comprising 1230 up-regulated and 24 down-regulated genes, into 3 databases (L1000FWD, DGIdb, and CMap) to identify significantly associated small-molecule compounds. The analysis revealed 92 compounds in the L1000FWD dataset, 3452 in DGIdb, and 16 in CMap. A Venn diagram showed 1 overlapping small-molecule drug between the CMap (n = 15) and DGIdb (n = 3345) datasets, and 6 overlapping drugs between DGIdb (n = 3345) and L1000FWD (n = 86). However, no compounds were common across all 3 datasets (Fig. 6A). For the CPTAC-2 dataset, 93 small-molecule drugs were identified in the L1000FWD dataset, 907 in DGIdb, and 366 in CMap as potential targeted drugs. The Venn diagram indicated 4 overlapping compounds across 2 of the databases, with 1 compound overlapping across all 3 datasets. Overall, we identified 12 compounds common across 2 databases: chlorhexidine, clomifene, cyproheptadine, doxorubicin, paclitaxel, mitomycin-C, alprazolam, digoxin, oxymetazoline, puromycin, simvastatin, and nadolol (Fig. 6B). These findings suggest potential small-molecule drugs that could be explored further for targeting CRC stemness traits.

## 4. Discussion

Despite significant advancements in CRC diagnosis and treatment over the past decade, the prognosis for CRC patients remains poor. Issues like drug resistance, chemotherapy resistance, and tumor recurrence highlight the limitations of current treatment strategies. A deeper understanding of the molecular and cellular heterogeneity in CRC could facilitate the development of new therapeutic approaches. According to the CSC hypothesis, cancer cells may originate from SCs, which play crucial roles in tumor progression and recurrence. Consequently, targeting CRC SCs could be essential for improving patient outcomes. In this study, we conducted a comprehensive analysis of CRC stemness using gene expression data from a large number of samples across 2 distinct CRC databases. We developed a stemness index model through an innovative OCLR machine learning approach, establishing an independent molecular metric of stemness. Our findings demonstrate that mRNAsi values in CRC are closely associated with CSC-related clinical characteristics. We identified 10 key genes with negative correlations to mRNAsi values, and we evaluated their prognostic potential using the Kaplan–Meier plotter database. Additionally, we utilized the L1000FWD, DGIdb, and CMap databases to screen for potential drugs that could target anticancer therapies. Our findings offer insights that may serve as a foundation for exploring the underlying molecular mechanisms of CRC and developing more effective treatments.

Survival curves were plotted to examine differences in PFS and DSS between the mRNAsi-H and mRNAsi-L groups. The results indicated that the mRNAsi-H group consistently exhibited better PFS and DSS compared to the mRNAsi-L group. This finding aligns with the work of Malta et al., who used Cox proportional hazards model analysis to demonstrate that a higher stemness index is associated with a better prognosis in CRC. Recognizing that the mRNAsi value represents various cell types within a sample, we adjusted the mRNAsi values by tumor purity and observed similar results, suggesting that the OCLR-derived stemness index is cancer-type dependent. Although vascular invasion is known to be significant in cancer progression,^[26]^ no difference in mRNAsi was observed between different vascular invasion states in CRC samples in this analysis. We also investigated the correlation between mRNAsi values and molecular subtypes in CRC samples. The results showed that mRNAsi values were significantly higher across the 4 CMS molecular subtypes than in normal samples. Additionally, the CMS4 subtype had notably lower mRNAsi values compared to the other CMS subtypes and was associated with the worst survival outcomes, as validated by the Kaplan–Meier method, consistent with previous studies.^[27]^ Interestingly, the CMS1 subtype tumors exhibited the highest stemness in both the TCGA-COAD and CPTAC-2 datasets. The CMS classification framework emphasizes distinct mechanisms for each subtype: nearly all hypermutated MSI tumors fall within CMS1, while CMS2 and CMS3 subtypes are marked by epithelial characteristics. When we combined stemness indices, we found that samples with a hypermutated phenotype had significantly higher mRNAsi values than those with EMT and epithelial phenotypes. These findings suggest that the stemness indices derived from the OCLR method are largely consistent with the widely accepted CMS molecular subtypes of CRC. Furthermore, studies have shown that mRNAsi can predict cancer recurrence and drug resistance.^[5]^

The Gene function annotation and KEGG pathway enrichment analysis were performed to facilitate further research. Functional annotations revealed that the DEGs were mainly enriched in the ECM, cell-substrate adhesion, and cell junction organization, suggesting potential roles in influencing tumor stemness by regulating SC self-renewal and differentiation.^[28]^ Tumor invasion and metastasis are complex, cyclic processes mediated by adhesion molecules. Pathway enrichment analysis showed that DEGs were predominantly associated with focal adhesion, the PI3K-Akt signaling pathway, and ECM-receptor interactions. Supporting these findings, previous studies have established a theoretical basis for CRC prognosis. For instance, PZR has been shown to accelerate CRC cell invasion and migration by enhancing FAK phosphorylation.^[29]^ Additionally, HIF-2α has been implicated in activating CSCs in breast cancer through the PI3K/AKT/mTOR pathway.^[30]^ Increasing evidence also suggests that ECM proteins create a biochemical and physical niche for CSCs, further promoting tumor progression.^[31]^ Together, these findings emphasize the impact of specific genes on the “stemness” phenotype of cancer, providing valuable insights for developing targeted therapies to effectively eliminate CSCs.

Ten key genes associated with CRC stemness were identified as differentially expressed between mRNAsi-L and mRNAsi-H groups, demonstrating strong inter-gene correlations. In line with enrichment analysis, 2 of these key genes, COL1A1 and COL1A2, are vital members of the collagen family, which is a crucial structural component of the ECM.^[32]^ Giampieri et al^[33]^ reported that COL1A1 can serve as a CSC differentiation marker, helping to predict relapse in resected CRC. Additionally, studies have linked COL1A1 upregulation in CRC with lymphatic metastasis, serosal invasion, and hematogenous spread.^[34]^ Bioinformatics analyses further suggest that homologous collagen family proteins, such as COL1A2 and collagen, type V, alpha 1, are highly expressed and associated with poorer DFS in CRC.^[35]^ Matrix metalloproteinases play crucial roles in tumor invasion, metastasis, and ECM degradation. Studies have shown that incubating CSCs with melatonin leads to a marked reduction in the expression and activity of matrix metalloproteinase 9. Additionally, umbilical cord mesenchymal stem cells have been shown to suppress matrix metalloproteinase and matrix metalloproteinase 9 overexpression, potentially inhibiting prostate cancer cell proliferation and invasion.^[36]^ BGN, another ECM protein, has been implicated in chemotherapy resistance in colon cancer through NF-κB signaling activation.^[37]^ The EMT-associated gene FN1 (fibronectin) is linked to a CD44^high^/EGFR^low^ phenotype, previously associated with stemness.^[38]^ Fibrillin 1 has also shown promising prognostic value for patients with early-onset CRC.^[39]^ Osteopontin (secreted phosphoprotein 1), an essential ECM protein, is involved in pathophysiological processes like cancer metastasis and may have diagnostic value in differentiating CRC lesions.^[40]^ Lastly, elastin, another ECM protein, has been shown to promote the proliferation of colon cancer epithelial cells.^[41]^ These 10 key genes offer valuable insights into the stemness and aggressiveness of CRC, highlighting potential biomarkers and therapeutic targets to improve CRC prognosis and treatment.

We queried the L1000FWD, DGIdb, and CMap databases using gene expression profiles from CRC samples with high and low mRNAsi levels. This analysis identified several compounds with known effects on CSCs (CSCs) in other tumor types, despite the limited number of treated cell lines represented in these databases. Notably, these compounds include the cytotoxic anthracycline antibiotic-Doxorubicin,^[42,43]^ the natural broad-spectrum antitumor drug-Paclitaxel,^[44]^ the cytotoxic antibiotic-Mitomycin-C,^[45]^ and the aminonucleoside antibiotic-Puromycin.^[46]^ Additionally, both Mitomycin-C and Puromycin have demonstrated anticancer effects on CRC cells, although their impacts on CRC SCs specifically have not yet been established.^[47]^ Importantly, research has shown that Doxorubicin can be conjugated with a CSC-targeting EpCAM aptamer to eliminate CSCs in CRCs.^[48]^ Paclitaxel has also been validated to reduce cancer malignancy by targeting CSCs when used in combination therapies.^[49]^ These compounds identified in our analysis may provide promising avenues for specifically targeting CRC SCs. Given that survival rates for CRC patients receiving nonspecific therapies have plateaued, integrating CSC-targeted therapies with conventional multimodal approaches may contribute to more durable CRC remission.

Since much of the data in the article is sourced from specific datasets, it is often limited to particular geographic areas, age groups, or ethnicities, making it challenging to generalize findings to broader populations. Big data analyses yield results at the group level, whereas clinical trial data offer insights into variability across individual patients, supporting the development of more personalized treatment options. In bioinformatics analysis using databases, the absence of clinical trial data can limit the interpretation and application of research findings, as these databases typically lack detailed clinical patient information, particularly regarding treatment responses, drug side effects, and long-term follow-up data. Clinical trial data, on the other hand, can provide real-world observations of disease progression and treatment efficacy, thus validating hypotheses generated from bioinformatic analyses. To enhance the clinical relevance of such analyses, it is recommended to integrate clinical trial data with bioinformatics results. Further experimental and biological validation is essential to substantiate the insights gained from our bioinformatics analysis. This research not only enhances our understanding of CRC stemness but also opens new avenues for targeted cancer therapies.

## 5. Conclusions

In conclusion, this study presents a thorough exploration of stemness in CRC, demonstrating a strong correlation between mRNAsi values and CSC characteristics through advanced machine learning techniques. We identified 10 pivotal genes that are crucial to CRC stemness, underscoring their promise as novel therapeutic targets for innovative treatments. Additionally, we uncovered several promising small-molecule drugs that have the potential to synergize with conventional therapies, paving the way for more effective treatment strategies. The implications of these findings are significant; however, further experimental and biological validation is essential to substantiate the insights gained from our bioinformatics analysis. This research not only enhances our understanding of CRC stemness but also opens new avenues for targeted cancer therapies.

## Author contributions

**Conceptualization:** Jing Lu.

**Writing—original draft:** Jing Lu.

**Data curation:** Haotian Zhang.

**Formal analysis:** Haotian Zhang.

**Investigation:** Xiaoyu Gu.

**Software:** Xiaoyu Gu.

**Methodology:** Yonghui Liu.

**Validation:** Chengwen Zhao.

**Funding acquisition:** Xudong Wang.

**Supervision:** Xudong Wang.

**Writing—review & editing:** Xudong Wang.

## Supplementary Material

**Figure:**